# Needs assessment of nurse researchers through a research lifecycle framework

**DOI:** 10.5195/jmla.2022.1461

**Published:** 2022-07-01

**Authors:** Robert Janke, Kathy Lynn Rush, Katherine Miller

**Affiliations:** 1 robert.janke@ucb.ca, Associate Chief Librarian, University of British Columbia, Okanagan Campus Library, Kelowna, BC, Canada.; 2 kathy.rush@ubc.ca, Professor, School of Nursing, University of British Columbia, Okanagan Campus, Kelowna, BC, Canada.; 3 katherine.miller@ubc.ca, Nursing Librarian, Woodward Library, University of British Columbia, Vancouver Campus, Vancouver, BC, Canada.

**Keywords:** libraries, medical, librarians, faculty, nursing, nursing research

## Abstract

**Objective:**

Health sciences librarian roles are evolving to better meet the needs of faculty. This study explores nursing faculty needs at the University of British Columbia through the research lifecycle framework of planning, conducting, disseminating, and assessing the impact of their research.

**Methods:**

A mixed methods survey study with Likert scale, multiple-choice, or ordinal ranking-scale questions and six open-response questions was conducted. The format was a web-based Qualtrics survey; participants had approximately three weeks to respond.

**Results:**

Nursing faculty identified the dissemination phase as benefiting most from library support prioritizing reference management and archiving research data as the top needs in that phase. Assessing impact skills such as citation analysis and Altmetrics training was ranked second. The Planning phase was ranked third with systematic review and literature review support most needed. The Conducting phase was identified as the phase where they needed the least support.

**Conclusion:**

Understanding the needs of researchers and enhancing scholar productivity is vital to offering responsive library research services. Across the research lifecycle, nursing faculty identified reference management, data management, metrics evaluation, systematic reviews, and literature reviews as the key areas for which they need support.

## INTRODUCTION

Liaison librarianship has evolved in the last 20 years from the traditional roles on the reference desk, as bibliographer and instructor. The traditional roles are in flux and face threats in the forms of invisibility and disconnection [1]. Kenney argues that in order to combat this invisibility and disconnection, librarians need to shift from being library or librarian-focused and instead develop engagement strategies based on the needs and success indicators of faculty [2]. Building on this idea, Jaguszewski and Williams suggest that the liaison model should be expanded to an engagement model whereby engaged liaisons aim to enhance scholar productivity and empower learners through participating in and supporting the entire lifecycle of research, teaching, and learning processes [3].

One way for libraries to support the entire research lifecycle is to develop or strengthen services aligned with the stages based on the needs of researchers. In a practical sense, Vaughan et al. explored whether pre-existing niche services of individual subject librarians could be marshalled into a suite of services available to all researchers across a university and throughout the research lifecycle [4]. From a more theoretical perspective, Maxwell was one of the first scholars to argue that libraries, and by extension librarians, need to organize themselves around the research lifecycle to strengthen or develop services that are in alignment with the university's research mission [5]. He argued this strategy would be important to show relevance and strategically important for the library to serve the needs of researchers in a research-centric way [5]. Maxwell acknowledges that on large university campuses there may be other departments offering support for specific aspects of the lifecycle and, in those cases, it is in the interest of the library to identify and develop strategic partnerships [5].

Biomedical and health libraries, and librarians, have a long history of conducting needs assessments of their patrons to determine where the library can best support researchers. Building on Maxwell's work, Ragon interviewed biomedical researchers who described their research activities under the conceptual categories of planning, conducting, disseminating, and assessing the impact of research [6]. What emerged was a rich collection of research activities under each of these categories and the proportion of library support those researchers used for each stage. Extending this work, He et al. published one of the few library science investigations into the needs of researchers along the continuum of the research lifecycle [9]. Although they used the research cycle proposed by Maxwell as a framework they chose not to investigate supports at the “project” or “conducting” phase. Instead, they focused on funding/grant application dissemination, and research impact assessment stages [9]. In contrast, Joo and Peters focused exclusively on the assessment of user needs for research data services (RDS) [10]. Broadly speaking, RDS are one of the ways that academic libraries support aspects of the conducting phase and can involve support for various data-related research activities from collection, to cleaning, to analysis, to visualization, to management. RDS are beginning to emerge in response to the growth of data-intensive research, changing the roles of libraries and the recognition of a need for data management [11].

**Figure 1:**
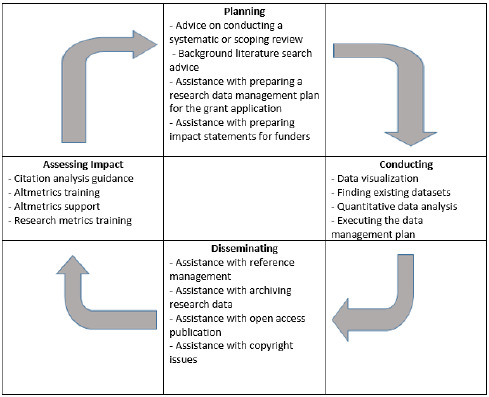
The Research Lifecycle as defined by Ragon with selected services.

This study contributes to this body of research, by building on the work of He et al. and Joo and Peters by uniquely assessing nurse researcher needs at each stage of the research lifecycle in its entirety as defined by Ragon (Figure 1). Specifically, our research questions are:

1. What are the perceived needs among nursing researchers as they pertain to the following stages of the research lifecycle: planning, conducting, publication, and assessing impact?
2. How do nursing researchers currently use library research tools and seek assistance for their research activities at each of the stages identified above, especially in light of the impacts of the COVID-19 pandemic?

## METHODS

### Instrument

Using the research cycle tested by Ragon [6] we designed a survey adapted from previously published research in this area [9, 10] to collect information about the perceived needs of nursing researchers [name and location of institution redacted for blind review], during the four stages of the research lifecycle: planning, conducting, dissemination, and assessing impact. The draft version of the survey was initially created by the principal investigator and subsequently reviewed by the other members of the team to reach agreement on the questions and to provide feedback to ready it for migration into Qualtrics. The survey was then pretested by the nursing faculty member and the second nursing librarian on the research team for question clarity, question order, and time to complete the survey and finalized for administration.

### Procedures

In advance of distributing the survey, the Behavioural Research Ethics Board at the University of British Columbia was consulted to see if this project required ethics approval. After reviewing a description of the project, it was determined that in accordance with the regulations of the Tri-Council Policy Statement governing research ethics in Canada, this study did not need to undergo ethical review as it was not classified as research.

An email distribution list for the survey was created by reviewing online employee directories from both Schools of Nursing. Because the focus of the survey was on nursing researchers, we did not recruit participants from the ranks of lecturers, emeritus faculty, or clinical associates and assistants for whom research is not an expectation of their roles. A final list of 112 names and emails was loaded into Qualtrics from which we could send the survey invitation as well as reminders. Qualtrics tracks who has completed the survey, and as a result does not needlessly remind individuals who have already completed the survey. During the three-week survey period, beginning April 26, 2021, two reminders were sent one week apart. To recognize the effort and time commitment of survey respondents two $50 gift certificates were offered as incentive prizes.

From the initial list of 112 invitees, Qualtrics identified two email addresses on the University of British Columbia email servers that were no longer reachable. As a result, the final population pool from which the sample was recruited was 110. The Data & Analysis and Reports modules within Qualtrics were used to descriptively analyze the responses to the quantitative questions. The PI and nurse faculty member thematically analyzed the results from the six open-ended questions independently. The identified themes for each question were compared and agreement was reached between the two analyzers.

## RESULTS

### Demographics, Assistance, and Workshops

When the survey closed we had received 30 completed responses, representing a 27.3% response rate. Respondents were at a variety of stages in their careers. In terms of years of research experience post degree, 22.6% (n=7) indicated 0-5 years, 19.5% (n=6) indicated 6-10 years, 16.7 % (n=5) indicated 11-20, and 38.7 % (n=12) indicated more than 20 years.

In the past year, 70% (n=21) of respondents indicated they sought help from the library to support their research. When asked to select the modes that they had used to seek help, the majority of respondents asked one of the Nursing Librarians (39.4% (n=13)) and/or another library employee (18.2% (n=6)) for assistance while 27.3% (n=9) indicated that they had accessed the library website. Popular sources of assistance for those that indicated that they did not seek assistance from the library, included colleagues in their department (31.8% (n=7)) followed by websites external to the University of British Columbia (22.7% (n=5)). The impacts of the COVID-19 pandemic on needs for library support were minimal. Overall, researchers (n=24) felt the pandemic had not changed their need for, or the library's ability to provide, that support. One participant explained that they “no longer physically needed to go to the library anyhow and all my exchanges with librarians and library staff were by email.” Generally, 73.3% (n=22) of faculty had not noticed increased, or a change in, needs for library support among their graduate students because of the pandemic as, in their opinion, the “library was already providing excellent support.” They also noted the need for more virtual support, greater access to electronic books, and a desire to continue to bolster the collection in that format.

Interest in attending workshops designed to support researchers was high with 72.4% (n=21) interested in attending, 17.2% (n=5) not interested in attending, and 10.3% (n=3) unsure, with one respondent choosing not to respond to the question. The preferred format for these workshops was live webinars (synchronous) with 57.1% (n=12) followed by equal preferences (19%) for in person (“when safe”) and recorded asynchronous videos. The eight individuals who had no interest or were unsure about workshop attendance had variable reasons for their responses: perceived self-competency with using the library or being too busy to fit them in. However, some faculty identified specific topics for which workshops would be of greater interest, such as conducting searches and systematic reviews.

### Research Lifecycle

Perceived need for support for the various stages of the research lifecycle was measured through a general question asking at which of the four broad stages researchers felt they could benefit from more assistance from the library and campus partners. Table 1 shows respondents' descending rankings (mean score along with the standard deviation (S.D.)) for the four stages of the research lifecycle in terms of level of perceived need for assistance (4=most assistance; 1=least assistance).

**Table 1:** Lifecycle stages ranked by perceived need for assistance.

Rank	Lifecycle Stage	Mean	S.D.
1	Disseminating (writing, publishing, and preserving your outputs)	3.04	1.02
2	Assessing impact (citation metrics, altmetrics, and author profiling)	2.56	1.06
3	Planning (grant preparation, research methodology finalization, and identification of collaborators)	2.35	1.0
4	Conducting (data collection, management, and analysis)	2.32	1.26

### Planning

Respondents were asked to rank twelve different services in order of perceived value to them during the planning process. Table 2 displays the results of this question in descending rank order based on mean scores.

**Table 2:** Planning stage services ranked by perceived value.

Rank	Service	Mean	S.D.
1	Advice on conducting a systematic or scoping review	5.5	1.2
2	Background literature search advice	4.7	1.4
3	Assistance with preparing a research data management plan for the grant application	4.0	0.9
4	Assistance with preparing impact statements for funders	3.9	1.2
5	Reference management support	3.8	1.7
6	Assistance with remaining current with research topics in your field	3.7	1.8
7	Assistance with open access requirements for funders	3.6	1.3
8	Provision of information to assist you with choosing, or learning more about, a research methodology	3.5	1.8
9	Assistance with finding grant and funding opportunities	2.9	1.7
10	Assistance in the form of a mentorship program with successful grant seekers	2.8	1.1
11	Provision of general funder policy guidelines	2.6	1.4
12	Assistance with identifying possible research collaborators	2.3	1.7

Respondents were also asked to select from a list indicating which profiling tools and reference management software they used (respondents could select multiple if they used more than one). The most popular profiling tool was ORCID 27.8% (n=20), followed by Google Scholar Profile 22.2% (n=16), and ResearchGate 20.8% (n=15). Two individuals used Scopus, but that was not available through institutional subscription at the time of this investigation. The most frequently used reference management tools were RefWorks and EndNote which were each selected by 29.6% (n=8) respondents.

### Conducting

Perceived need for support with data-related processes in the conducting phase of the research lifecycle were scored on an ordinal scale from 1=”not at all needed” to 5=”extremely needed.” Table 3 displays the various processes in rank order from most needed to least needed.

**Table 3:** Data-related conducting stage processes ranked by level of perceived support.

Rank	Aspects of Conducting Research	Mean	S.D.
1	Data visualization	3.4	1.2
2	Finding existing datasets	3.2	1.3
3	Quantitative data analysis	2.8	1.5
4	Executing your data management plan	2.7	1.3
5	Data refinement or cleaning	2.6	1.3
6	Data collection	2.4	1.4
7	Qualitative data analysis	2.2	1.3

In addition to data related processes, we also investigated the level of activity with, and the perceived need for support for, systematic or scoping reviews. Researchers were engaged to varying degrees with knowledge synthesis (e.g. systematic or scoping reviews) with 10 actively working on a systematic or scoping review, 10 planning to work on one in the coming year, and 10 who had completed a review in the past year. A limitation of the wording of the survey was that there may be overlap in these responses. Sixteen researchers had one or more graduate students doing a systematic or scoping review. Seventeen respondents indicated that for these activities they had consulted a librarian, while 5 indicated that a librarian was on their research team and recognized as a co-author; a further 18 said that they have recommended that their graduate students consult with a librarian on their reviews. Faculty respondents praised the library's support in their open text comments, such as assistance is “always helpful and excellent” and the “introductory library presentation [is] very valuable for first term graduate students.” One participant thought, “it would be great to have a librarian as a co-author” and that they “haven't done so yet but support it.”

### Disseminating

Respondents reported the dissemination phase as the one where they needed most assistance when compared to the other three stages of the research life cycle. Within this phase, assistance with reference management and archiving research data were among the activities ranked as most valuable (Table 4).

**Table 4:** Dissemination stage services ranked by perceived value.

Rank	Service	Mean	S.D.
1	Assistance with reference management	3.3	1.1
2	Assistance with archiving your research data	3.0	1.1
3	Assistance with open access publication	2.8	0.9
4	Assistance with copyright issues	2.5	0.9
5	Assistance with archiving publications	2.5	0.9
6	Assistance with identifying publication venues	2.4	1.2
7	Assistance with meeting funder mandates and/or requirements	2.3	1.1
8	Assistance with negotiating licenses	1.8	0.9

When nurse researchers were asked to select service options that they would engage in for improving their research manuscripts, 50% (n=13) identified access to a copyediting service from a writing professional, followed by interest in a professional development course with colleagues (15.4%; n=4) and one-on-one assistance with a writing professional (3.9%; n=1). Six individuals indicated that they would not engage in any of the options listed and did not indicate any other options.

The majority of respondents indicated that they either occasionally (50%; n=13) or regularly (38.5%; n=10) used open access publishing models. The survey did not specify which models but instead used open access in the broadest sense. As a result, respondents could be engaging in a range from institutional repositories to author publishing charges (APCs). A minority 7.7% (n=2) of researchers acknowledged that they were not familiar with open access while 3.8% (n=1) indicated they were aware of open access publishing models but had not employed them in their own research dissemination. A cross-tabulation (Table 5) to explore patterns of open access engagement by years of research experience revealed that, amongst researchers who had 0-5 years of experience, only 16.7% (n=1) regularly used open access models in comparison to 45.5% (n=5) of those with 20+ years of experience. Further, open access publishing was not the top journal characteristic influencing where respondents would publish, rather, top characteristics included academic reputation (n=7), impact factor (n=7), and area of coverage (n=6).

**Table 5:** Open access engagement by research experience.

		Experience as an academic researcher (years				
		0–5	6–10	11–20	20+	Total
Current engage ment with open access publica tion	I am not familiar with open access publishing models	1 16.7%	0	0	1 9.1%	2
	I am familiar with open access publishing models but do not employ them in my own research	1 16.7%	0	0	0	1
	I occasionally use open access publishing models for my own research	3 50.0%	3 60.0%	2 50.0%	5 45.5%	13
	I regularly use open access publishing models for my own research	1 16.7%	2 40.0%	2 50.0%	5 45.5%	10
	Total	6 (100%)	5 (100%)	4 (100%)	11 (100%)	26 (100%)

### Assessing Impact

Among the list of services providing support for, and activities related to, assessing research impact, respondents ranked citation analysis guidance highest at this stage as outlined in Table 6.

**Table 6:** Impact assessment stage services ranked by perceived value.

Rank	Service	Mean	S.D.
1	Citation analysis guidance	3.5	1.0
2	Altmetrics training	3.4	1.7
3	Altmetrics support	3.3	1.2
4	Research metrics training	3.3	1.2
5	Provision of research trend reports	3.2	1.3
6	Assistance with benchmarking at departmental and institution level	3.1	1.3
7	One-on-one consultations	2.9	1.7
8	Assistance with topical bibliometric/publishing trend analysis	2.5	1.5
9	Assistance with tenure and promotion profiles	2.4	1.4

The top needs in terms of specific areas of research impact assessment were both journal-based metrics (29.2%; n=7) and alternative metrics (29.2%; n=7) and was followed of individuals selecting that none of these areas were needed (16.7%; n=4). Somewhat fewer (12.5%; n=3) respondents selected either research output metrics or metric visualization. The most frequently used resources researchers used when conducting research impact measurement activities were Google Scholar metrics which was selected by 37.2% (n=16), followed by Journal Citation Reports (18.6%; n=8) and Web of Science (14%; n=6). Resources that were used less frequently included SciVal (4.7%; n=2), Impact Story, Publons, and Scimago which were each selected by 2.3% (n=1) of respondents. Twenty-seven percent of individuals indicated no use of resources and did not indicate other tools when given the option to elaborate.

### Needs, Emerging Trends, and Final Thoughts

Forty-seven percent (n=14) of faculty spoke of needs, emerging trends, or practices and their influence on research, with open access publishing a recurring area of interest for both themselves and their graduate students. Related, work on open platforms such as Open Science or making greater use of cIRcle, the University of British Columbia's institutional repository, to store data, preserve, and disseminate research outputs was also noted. There was also interest in exploring alternative ways of disseminating research findings/data and knowledge translation venues in alternative formats such as through data visualization (using GIS technology and other digital technologies) and presenting abstracts by video. One final theme was the acknowledgment of the growing importance of social media, not only for sharing research “efficiently and effectively” but also for “research impact activities.”

Themes that emerged in the nine responses regarding final thoughts related to library support for research revolved around the quality of support and specific suggestions for services. Faculty had suggestions for enhancing support, particularly of new faculty and graduate/doctoral students during orientation. Related to this was the importance of communicating library services to orient those new to the university. Assistance with impact metrics and a workshop covering Mendeley were also suggested. Faculty also used this as an opportunity to praise the library mentioning the “excellent support” they were receiving and how they “can't imagine how a university [teaching and research] would function in today's online world without the expert support of librarians.”

## DISCUSSION

This is the first study to assess the library support needs of nursing faculty at a large research-intensive Canadian university across the research lifecycle. While it shows the faculty's highest-ranked support needs related to dissemination services, they reported needs across the entire research cycle.

### Research Lifecycle

Nurse researchers prioritized the disseminating phase of the research lifecycle as their area of greatest need for library assistance and ranked the conducting phase as the area of least need. This is consistent with other research suggesting the conducting phase as weaker than other phases of library support although there are signs that this may be shifting as libraries begin to adapt [6]. As a result, researchers may need assistance at this stage but traditionally have not seen the library as a source of support. Research has found that health sciences faculty were familiar with the services available to them at the beginning and end of the research process but were surprised to learn that librarians can provide support throughout the entire research lifecycle [4].

### Planning

Previous research with faculty on supports during the planning phase ranked a mentorship program with successful grant seekers, and provision of general funder policy guidelines among the most beneficial potential research services [9]. Our findings did not replicate these. Instead, faculty in our investigation ranked those two services among the lowest in perceived need from a list of 11. Some reasons for this may be that other units such as the University's Office of Research Services offer these supports. Additionally, there are staff in the School of Nursing that may be already providing these services in an exemplary way. Faculty ranked advice on conducting a systematic or scoping review, an option not provided by He et al, as well as advice on background literature search among the areas where they feel a need for the most assistance. This reflects McKeown and Ross-White findings of increased requests from faculty to have a librarian conduct systematic review searches [12]. Other research has emphasized the importance of literature searching during the planning stage as a library support, especially as they relate to grant applications, to establish background to the project or to support the research methods chosen [13].

### Conducting

The research data services that ranked highest in terms of perceived need were data visualization and finding existing datasets. This contrasts with the results by Joo and Peters who found that among health science researchers at the University of Kentucky, assistance with qualitative data analysis and data management plans were ranked the highest [10]. Assistance with data analysis was also frequently mentioned in interviews with a multidisciplinary group of faculty at the University of Kansas [14]. In our study, data analysis and data management plans were among the lowest ranked items. Part of the explanation may be that data management plans are currently not required by the major research funders in Canada and that Joo and Peter's survey respondents were composed of over 50% graduate students, who may have different needs compared with the nurse researchers in our study, who were faculty members and post-doctoral researchers [10].

Current findings showed a relatively high level of nurse researcher engagement in systematic and scoping review with a third planning to work on one in the coming year and over half having their graduate students working on one and relying on librarians for assistance. Other researchers have also found a high degree of reliance on the library for assistance with systematic reviews (SRs) [15]. Within the Canadian context, the proportion of health sciences librarians who have participated on a SR in the past year is high (over 65%) with lack of time being the most frequently reported factor limiting the ability to assist with SRs [16]. Although nurse researchers in this survey appear to be satisfied with the current level of support with systematic reviews, the development of a more formalized service that supports them while addressing librarian workload issues, to combat high rates of burnout among information professionals who support SRs [17] would be beneficial while also identifying training needs among librarians and patrons alike [16].

In this study, for those nursing researchers who indicated they were engaged with doing reviews 14.3% (6) indicated that a librarian was on their research team as a co-investigator, but there was interest among faculty to have a librarian as a team member. Other research with nursing faculty published in 2009 reported a very small minority (8%) of them see librarians as partners in research pursuits and would be open to researching and/or publishing together as scholars [18]. Research has identified the mutual benefit for teams and librarians of having librarians embedded on research teams [19].

### Disseminating

At the disseminating phase, our results echo previous research which found that assistance with reference management, ranked as one of the most needed supports [9]. Cain et al. also noted that within their category of digital capture, publishing, and preservation, faculty showed a high demand for “enhanced support for manuscript and grant preparation,” which included an example that may imply reference management support¬ “writing and editing services” [8].

A relatively high proportion of respondents (88.5%) engaged to some extent with any type of open access publishing. This was a higher level of engagement when compared to other health science faculty in Canada (37%-57%) [9, 20] and the US (44%) [21] and to faculty from all disciplines (65.3%) [22]. This compares with results among dentistry faculty at the University of Toronto that found 57.1% occasionally or regularly used open access publishing models [9]. The most important reasons for choosing which journals to publish in among nursing researchers in this investigation were not dissimilar to those seen in other studies. Among this research and others, academic reputation and impact factor all ranked highly [9, 21]. Considering the high level of engagement with open access publishing among this population, it may be justified to begin to target educational interventions at a more nuanced level including the differences between pre-print and post-print, repository-based, and APCs.

Although a professional development program designed by librarians and instructional design staff that included access to nursing writers who shared their experiences appears to have worthwhile scholarship outcomes for nursing faculty [23], this activity measured in the current survey only garnered interest from a small number of faculty (15.4% or n=4). Instead, there appeared to be a much larger preference (50% of n=13) for access to a copyediting service from a writing professional.

### Assessing Impact

Similar to other research, research metrics training and citation analysis guidance were ranked as among the most important research impact assessment services [9]. Conversely, altmetrics training and support were ranked quite highly in our investigation but were ranked among the least important by respondents in a previous study [9]. These different rankings may stem from confusion or a lack of knowledge about altmetrics among faculty. In a US study, 71.4% of respondents indicated that they were either “not at all” or “marginally” familiar with “altmetrics” or non-traditional means of demonstrating scholarly impact [24]. Other research also revealed that there is a clear knowledge gap between librarians' knowledge of metrics compared to faculty and the need for librarians to fill this gap by providing metrics information to faculty [25]. Related to this are findings that what faculty want in terms of metrics are useful guides on how to find impact measures most relevant to their own field of study [24]. Similar to other research, nurse researchers tended to use Google Scholar metrics the most with Journal Citation Report and Web of Science distant second and third choices [24]. Other research with dentistry researchers noted the ascendency of Web of Science followed by Scopus and then Google Scholar [9].

### Workshop Interest and Assistance Seeking

In terms of workshops, 72.4% of respondents indicated an interest in attending them. This contrasts with past research with health sciences faculty that found the majority were neutral about the importance of library workshops, classes, or presentations in meeting their information needs [26]. Among respondents there was also a clear preference for synchronous or asynchronous online formats (76.1%) which is also supported by other research with nursing faculty pre-pandemic [23] and must be considered when planning workshops. To remain responsive to all patrons it will be important to gain a greater understanding of the 19% who indicated a preference for in-person (when safe) and any barriers to online delivery that might be addressed.

In response to the COVID-19 pandemic, the University of British Columbia library closed physical spaces and pivoted to supporting researchers and other patrons exclusively online, yet 70% of respondents from this survey indicated that they sought help from the library to support their research at least once in the past year of the pandemic. Further, of those who sought library support 37% (n=13) sought assistance from a nursing librarian. Similarly, in research done with health sciences faculty pre-COVID, assistance from library personnel was rated as very important (40%) or important (35%) in terms of library services [26]. Conversely, other research into help seeking behavior of faculty found that those in the natural and physical sciences did not typically seek out librarians [27]. Instead, research conducted with diverse academic units indicated a high likelihood that researchers may use their peer networks as their starting point when looking for help [28].

### Limitations

This study has several limitations including non-response bias, lack of generalizability, methodological limitations as well as the unknown impacts of the COVID-19 pandemic. The survey invitation subject line “University of British Columbia Library Support for Nursing Research Survey” may have recruited individuals more likely to have an opinion of the library or are library users. Although administered after the academic term to reduce competing teaching commitments there may have been other priorities such as grant applications and programs of research that may have hampered individuals from responding. Further, when responses were submitted there may have been inaccuracies and inconsistencies in the data because of the use of terms such as altmetrics and citation analysis that required a degree of interpretation on behalf of respondents. The small sample size, in part due to the exclusion of lecturers, clinical associates, and assistants, reduces generalizability of the findings. Another limitation to this study was the exclusion of graduate students. They were excluded for a number of reasons including: the fact that the majority of them, at least at the MSN level, do not engage in the thesis stream; their transitory nature at the institution; and because the library needs of active nurse researchers was the focus of this study.

The use of a survey with only a few open-ended questions limited qualitative feedback. However, responses highlighted areas for further study such as the impacts of COVID-19 on research needs, processes, and research impact assessment. This is clearly an avenue for further qualitative study. Another area for further inquiry would be to investigate nurse researchers in comparison to researchers from other disciplines. This would enable insights into the influence that a nursing scholarship might have, in contrast to other disciplines, on scholarly communication practices and interactions with librarians throughout the research lifecycle. Also, the needs of graduate student researchers would be another fruitful area for future research.

## CONCLUSIONS

We investigated the perceived needs of nursing faculty across the stages of the research lifecycle and explored opportunities for library support. Faculty identified the disseminating phase as the top ranked need and the conducting phase as the lowest ranked need, a finding that may reflect faculty perception of traditional but not emerging librarian roles. Within the planning phase, the need for advice on systematic or scoping reviews and background literature searching were highly ranked and the overall top ranked need of survey respondents. This is not unexpected given the librarian's expertise in these domains. During COVID-19, faculty continued to receive the same high level of library services as pre-COVID-19. Findings point both to the need to enhance faculty awareness of the full range of librarian roles, particularly as they continue to emerge, and to coordinate among other university services to avoid duplication. Librarians can help shape future directions for the library and advance the library's mission within the larger institutional context by investigating the evolving needs of researchers and students. Building strong, collaborative relationships with faculty and other campus professionals are necessary building blocks to ensuring librarians' future success and relevancy [3].

## Data Availability

Data associated with this article, including the results, survey instrument, and text of the invitation email are available in Scholar Portal Dataverse: https://doi.org/10.5683/SP3/OUJWXP.
